# Latent Classes of Polysubstance Use and Associations with HIV Risk and Structural Vulnerabilities among Cisgender Women Who Engage in Street-Based Transactional Sex in Baltimore City

**DOI:** 10.3390/ijerph19073783

**Published:** 2022-03-22

**Authors:** Sam Wilson Beckham, Jennifer L. Glick, Kristin E. Schneider, Sean T. Allen, Lillian Shipp, Rebecca Hamilton White, Ju Nyeong Park, Susan G. Sherman

**Affiliations:** 1 Department of Health, Behavior and Society, Bloomberg School of Public Health, Johns Hopkins University, Baltimore, MD 21205, USA; jglick5@jhu.edu (J.L.G.); sallen63@jhu.edu (S.T.A.); rhwhite.8500@gmail.com (R.H.W.); ju_park@brown.edu (J.N.P.); ssherman@jhu.edu (S.G.S.); 2 Department of Mental Health, Bloomberg School of Public Health, Johns Hopkins University, Baltimore, MD 21201, USA; kschne18@jhmi.edu; 3 Department of Epidemiology, Bloomberg School of Public Health, Johns Hopkins University, Baltimore, MD 21201, USA; lshipp2@jhu.edu

**Keywords:** substance use, sex work, structural vulnerabilities, latent class analysis

## Abstract

We describe patterns of polysubstance use and associations with HIV risk-related behaviors among women engaged in street-based transactional sex, an understudied yet important population and area of research. This sample was restricted to cisgender women who reported drug use (*n* = 244) in the baseline of the longitudinal SAPPHIRE cohort study. Latent class analysis (LCA) was conducted using drug use measures (route of administration (injection/non-injection); type of drug (specific opioids, stimulants)) and selection based on fit statistics and qualitative interpretation of the classes. Polysubstance use was prevalent (89% ≥ 2), and 68% had injected drugs in the past 3 months. A three-class solution was selected: Class 1 (“heroin/cocaine use”, 48.4% of sample), Class 2 (“poly-opioid use”, 21.3%), and Class 3 (“poly-route, polysubstance use”, 30.3%). Class 3 was significantly younger, and Class 2 was disproportionately non-White. Women reported high levels of housing (63%) and food (55%) insecurity, condomless sex with clients (40%), and client-perpetrated violence (35%), with no significant differences by class. Obtaining syringes from syringe services programs differed significantly by class, despite injection behaviors in all classes. Tailored HIV and overdose prevention programming that considers drug use patterns would strengthen their impact.

## 1. Introduction

Globally, women engaged in sex work have an enduring, elevated prevalence of HIV and sexually transmitted infections (STI), accounting for 15% of all HIV/AIDS cases and over 100,000 deaths due to HIV/AIDS [1,2,3]. While US population-level data on HIV prevalence among women engaged in transactional sex is limited [4,5], surveillance data among high-risk heterosexuals found a 4.1% HIV prevalence among the women who traded sex [6]. Studies have found HIV prevalence in the US ranging up to 17% among cisgender women engaged in transactional sex [1,4] and 5.2% in Baltimore, where the present study took place [7]. This indicates that this population is at a significantly elevated risk of contracting HIV compared to the general US population.

There is a myriad of structural and individual factors that drive elevated rates of HIV risk among women engaged in transactional sex. Structural determinants of heightened HIV risk include lack of access to economic opportunities, housing insecurity, food insecurity, violence, policing practices, and criminalization of sex work [7,8,9,10,11,12,13]. These broader structural dynamics strongly influence individual-level HIV risk factors and behaviors, including patterns of substance use.

Women who use drugs also experience specific risk factors and structural vulnerabilities that have important implications for their health and further compound their HIV risk [14,15,16]. We differentiate between risk (e.g., an individual behavior) and vulnerabilities (an “interactive process between the social context in which a … person lives and a set of underlying factors that … place the … person ‘at risk’ for negative outcomes”) [17] to acknowledge the environmental and structural factors that place women “at risk of being at risk”, instead of looking at individual behavior alone. Estimates of transactional sex among women who use drugs range from 15–66% in the US, and women often exchange sex to pay for drug use [16,18]. Women who use drugs and exchange sex have heightened HIV-related risks such as condomless sex, more partners, client/partner violence victimization, and syringe sharing, along with overall higher mortality rates [1,19,20,21,22,23]. Women’s occupational risk environments are compounded by the dual criminalization of sex work and drug use, leading to increased interaction with law enforcement and incarceration among women who sell sex [24]. Polysubstance use, defined in this manuscript as the use of multiple substances within a designated time (here, 3 months), but not necessarily at the same time, may further compound these risks. Polysubstance use in women particularly has been shown to be associated with an increased likelihood of violence, sexual risk behaviors, engagement in transactional sex, and poverty [25,26,27].

Studies have sought to examine these risk factors and structural vulnerabilities separately among people who use drugs [24,28,29,30,31,32] and women who exchange sex [33]. Although these behaviors often occur in tandem, few have employed latent class analysis (LCA) to examine the complex patterns of polysubstance use among women who exchange sex [27,34,35,36]. LCA has been used to understand patterns of polysubstance use of both injection and non-injection drugs among various populations, including adolescents [37], adults in urban and rural settings [38,39,40,41,42,43], and women living with HIV [26]. Classes of people who engage in polysubstance use have been shown to have a differential risk of poor health and Hepatitis C virus (HCV) [41,42]. They also have differing degrees of engagement with harm reduction strategies, such as possession of naloxone to prevent overdose fatalities [39], syringe acquisition at syringe services programs (SSPs), and interest in PrEP [44]. While people who use multiple substances through injection generally face greater health-related risks, these studies show that non-injecting classes also face unique risks and vulnerabilities that should be addressed [38,42]. Furthermore, while LCAs exploring substance use have found polysubstance use to be associated with sex work [26,42], we found few LCA studies exploring substance use specifically among women who sell sex [45,46].

Given the dearth in the literature on risks and vulnerabilities associated with polysubstance use among women who exchange sex [46], the aim of this paper is to describe patterns of substance use and associations with HIV-related risks and structural vulnerabilities among cisgender women engaged in street-based transactional sex.

## 2. Materials and Methods

### 2.1. Study Design

The Sex Workers and Police Promoting Health in Risky Environments (SAPPHIRE) study was a prospective cohort of cisgender (*n* = 250) and transgender (*n* = 62) women involved in street-based sex work in Baltimore City, Maryland. This is a secondary analysis of the baseline data of SAPPHIRE. The full study procedures and sampling methods are described elsewhere [7]. In brief, participants were recruited using targeted sampling in 14 locations known for sex work activities. Eligibility criteria were age ≥15, identified as a woman, sold or traded sex (oral, vaginal, anal) “for money or things like food, drugs or favors”, picked up clients on the street or public places ≥3 times in the past 3 months, and willing to undergo HIV and STI testing.

### 2.2. Analytical Sample

The analytical sample for this paper was restricted to cisgender SAPPHIRE baseline participants who indicated substance use (by any route of administration) in the past 3 months, yielding a final analytical sample of 244 women (97.6% of all cisgender participants). Due to the small sample size, low prevalence of substance use among the transgender women (*n* = 25), and different risks and vulnerabilities compared with cisgender women [7], we conducted the LCA using only the cisgender portion of the sample.

### 2.3. Measures

#### 2.3.1. Latent Class Indicators

Past 3-Month Substance Use. We included measures of substance use by both the type of drug used and route of administration in the past 3 months, which we will refer to hereafter as “recent” substance use. For drugs that can be obtained by prescription, we considered only drug use not prescribed by a clinician. The following measures informed the class model: heroin (injection), cocaine (injection), heroin and cocaine co-injection (speedball, injection), opioid pain relievers (sniffed/snorted, swallowed), buprenorphine or Suboxone (snorted/sniffed, swallowed), heroin (smoked, snorted/sniffed), crack or powder cocaine (smoked, snorted/sniffed), heroin and cocaine together (snorted/sniffed), and benzodiazepines (snorted/sniffed, swallowed). Due to the low prevalence of recent use, we excluded ecstasy/MDMA and prescription stimulants (i.e., Ritalin, Adderall) from the model.

#### 2.3.2. Covariates

Sociodemographic Characteristics. Age: in years, was retained as a continuous covariate. Race/ethnicity: Women were asked to select all that apply for their race and ethnicity (with options to specify as well). Due to the small sample size of some non-White and non-Hispanic groups, we dichotomized race/ethnicity to (1) non-Hispanic White, and (2) Black, Indigenous, and People of Color (BIPOC). Education: Education was dichotomized as less than high school education versus high school graduate, obtained GED, or higher.

#### 2.3.3. Outcomes

Outcomes were selected to represent risks and vulnerabilities [17] at interpersonal (condomless sex, client violence) and structural levels (housing and food insecurity, accessing syringe services programs). *Condomless transactional sex* was defined as not always using a condom during any vaginal or anal sex with clients (yes/no). *Client violence* was a combined, dichotomous measure of client-perpetrated violence from two variables: physical violence (being hit, punched, slapped, threatened, or having had a weapon used against their person (Revised Conflict Tactics Scale) [47]) and sexual violence (forced or pressured sex) (yes/no). *Housing insecurity* was ascertained by asking if women had experienced homelessness (yes/no). *Food insecurity* was defined as going to bed hungry at least once per week because there was not enough food (yes/no). *Obtaining syringes from an SSP* was defined as accessing syringes through an SSP vs. only elsewhere (e.g., pharmacy) (yes/no). All outcomes were reported in the past 3 months.

### 2.4. Analysis

A latent class analysis was conducted using substance use variables to identify profiles of polysubstance use. LCA uses indicators (i.e., specific substances) to identify probabilities of membership in latent, homogenous groups of participants based on their drug use profiles. Models with one through five classes were estimated using a full information maximum likelihood estimator. In the case of missing data, this method uses all available data from each case to estimate missing data using an imputation-like procedure [48]. To determine the final number of classes, we estimated and considered various model fit statistics (Akaike information criterion (AIC), Bayesian information criterion (BIC), and Lo–Mendell–Rubin likelihood ratio tests (LRT)), and qualitatively interpreted the classes [49].

After the selection of a three-class model, we estimated the probabilities of class membership and the conditional probabilities of each indicator on class membership. We employed the R3STEP approach to assess unadjusted and adjusted associations between sociodemographic variables and class membership [50]. When testing associations between identified latent classes and the outcomes, we used the BCH method to apply weights that adjust for potential misclassification of participants into classes [51]. Sociodemographic correlates that were associated with class membership significant at the *p* < 0.05 level in the unadjusted analysis were included in the multivariable outcome models [51]. We used Wald tests to determine overall differences in expected values of distal outcomes between the classes and then used pairwise tests with additional model constraints, with significance measured at the <0.05 level. We used Mplus 8.4 for LCA analyses.

### 2.5. Ethical Statement

The study was approved by the Johns Hopkins University Bloomberg School of Public Health Institutional Review Board (protocol number IRB00005939). All participants gave informed consent before participation and received a $70 USD VISA gift card for completing the baseline visit.

## 3. Results

The majority (67%) of participants were non-Hispanic White (Table 1). The mean age was 35.8 years, and about half (52%) had less than a high school education. Further details of the sociodemographics of the sample can be found elsewhere [7]. Almost all (88.9%) of the sample reported recently using ≥2 substances, and two-thirds of the sample reported recent injection drug use (68%). Injected heroin was most common (67%), followed by injected cocaine (25%) and injected heroin and cocaine together (23%). Non-injection drug use was nearly universal in this sample (98%). A majority of all the women used cocaine or crack (87%); many (43%) snorted, sniffed, or swallowed heroin; and some (9%) used heroin and crack/cocaine simultaneously. The use of benzodiazepines (30%), opioid pain relievers (29%), and buprenorphine/Suboxone (17%) were comparatively less frequent.

Study participants reported high levels of food and housing insecurity (52% and 61%, respectively). Condomless sex with clients was common (40%), as was client-perpetrated physical and sexual violence (35%). Many participants (43%) reporting obtaining syringes from only SSPs vs. elsewhere in the past 3 months.

### 3.1. Latent Classes of Polysubstance Use

A three-class solution was selected based on fit statistics and substantive interpretation of the classes (Table 2).

Table 3 reports item–response probabilities of the drug use indicators by the three classes and the proportions of the sample based on posterior probabilities of most likely class membership. These are also displayed graphically within Table 3; a visual representation of most likely class membership is helpful to see the different class patterns of substance use. Class 1 (“heroin/cocaine use”) was the largest class (48.4%) and was characterized by the use of heroin by injection (58% probability) and non-injection (40% probability), non-injection crack/cocaine (87% probability), and little or no use of other substances (i.e., no opioid pain relievers, <14% probability of benzodiazepines, <5% probability of buprenorphine). Class 2 (“poly-opioid use”) was the smallest class (21.3%) and was characterized by the use of non-injection heroin (58% probability) and cocaine/crack (71% probability), but usually not at the same time (11% probability), and higher use of opioid pain relievers (75% probability), benzodiazepines (55% probability), and buprenorphine/Suboxone (26% probability) compared with any other class. This class had the lowest probability of injecting heroin (39%) and did not inject other substances. Class 3 (“poly-route, polysubstance use”) (30.3% of the sample) was characterized by high proportions of all routes and substances, especially injection. This class was also characterized by simultaneous use of heroin and cocaine by both injection (72% probability) and non-injection (42% probability).

### 3.2. Sociodemographic Correlates of Latent Class Membership

Table 4 summarizes the distribution of demographic characteristics by most likely latent class membership. Class 1 heroin/cocaine use was on average older than Class 2 poly-opioid use but not significantly (mean 37.3 vs. 35.5 years). However, Class 3 poly-route, polysubstance use was significantly younger than Class 1 (mean 34.0 vs. 37.3 years; β = −0.041, *p*-value = 0.038). Race/ethnicity differed significantly across class membership. BIPOC women comprised 31% of Class 1 heroin/cocaine use, 48% of Class 2 poly-opioid use, and only 24% of Class 3 poly-route, polysubstance use. These proportions were significantly different between Class 2 poly-opioid use and Class 1 heroin/cocaine use (β = 0.851, *p*-value = 0.046) and between Class 3 poly-route, polysubstance use and Class 2 poly-opioid use (β = −1.09, *p*-value = 0.012). Educational level did not differ significantly across classes, whereby 51% of Class 1 heroin/cocaine use, 45% of Class 2 poly-opioid, and 59% of Class 3 poly-route, polysubstance use had less than a high school education.

### 3.3. HIV-Related Risks and Structural Vulnerabilities by Latent Class

**Figure 1:**
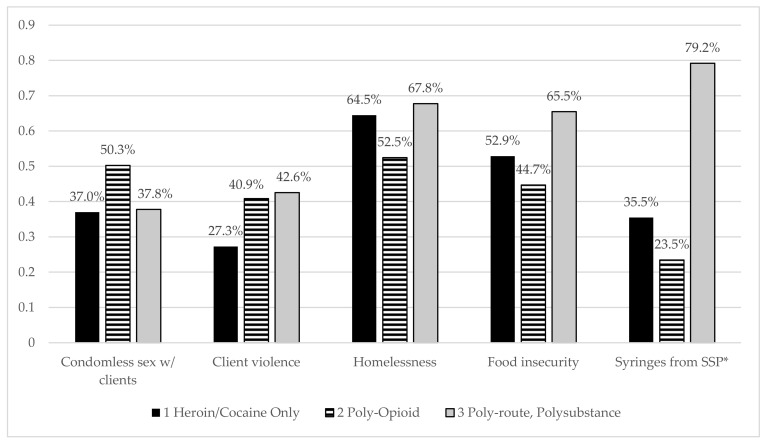
Prevalence of outcomes by substance use latent class, controlling for age and race; * Wald test significant at <0.05 level.

The probabilities of five different structural vulnerability and HIV-related risk outcomes by class are shown in Figure 1, controlling for age and race/ethnicity. There were no significant differences in recent condomless vaginal/anal sex with clients between classes. Class 1 heroin/cocaine use had a lower prevalence of recent experience of client physical or sexual violence (27%) than the other classes (41% in Class 2 poly-opioid use and 42% in Class 3 poly-route, polysubstance use), but not significantly. Housing insecurity was high in all classes, but not significantly different between classes, with housing insecurity experienced by 65% of Class 1 heroin/cocaine use, 53% of Class 2 poly-opioid use, and 68% of Class 3 poly-route, polysubstance use. Prevalence of food insecurity was highest among Class 3 poly-route, polysubstance use (66%), but not significantly different from Class 1 heroin/cocaine use (53%) or Class 2 poly-opioid use (45%). Obtaining syringes from SSPs differed significantly by class (Wald *p*-value < 0.000): 36% of Class 1 heroin/cocaine use, 23% of Class 2 poly-opioid use, and 79% of Class 3 poly-route, polysubstance.

## 4. Discussion

This study was one of the first to employ latent class analysis to examine the unique patterns of polysubstance use among a sample of women engaged in street-based transactional sex in a US city. Findings provide a nuanced understanding of the nature of these women’s drug use patterns as well as associations with prevalent structural vulnerabilities and HIV risks. We identified three classes with distinct polysubstance use patterns considering specific substances and routes of administration: Class 1 primarily used injection heroin and non-injection cocaine/crack, Class 2 primarily used injection and non-injection opioids, and Class 3 used all measured substances. Further, we found that HIV risks and vulnerabilities were high in all classes, while differences dependent on class membership have practical implications for future research and tailored interventions.

The HIV risk outcomes (condomless sex with clients, client-perpetrated violence, homelessness, food insecurity) were high across all classes. While there were some differences by class in our analysis (i.e., Class 2 was less likely to use condoms and experience homelessness and food insecurity, Class 1 experienced less client violence), these associations were not significantly different by class. Our findings differ from an LCA of women living with HIV in Canada that found higher levels of violence and lower-income status among women in polysubstance use classes compared with women in single-use or non-use classes [26]. It is possible that our findings differ because all classes indicated polysubstance use and, therefore, experienced higher risks overall; our sample was comprised of women who both sold sex and used drugs and are, therefore, multiply marginalized. This may also be due to a lack of power to detect differences in these outcomes. Our findings add support to the literature that shows that the factors contributing to the HIV risk environment impact women across a variety of polysubstance use classes. Given the high prevalence of HIV-related risks across all classes, prevention interventions should target women who sell sex and use drugs of all types, not only women who inject.

Use of SSPs did differ significantly by class. Importantly, the members of Class 3 poly-route, polysubstance use were more likely to access syringes at SSPs vs. other locations (e.g., pharmacy). It is encouraging that women who inject multiple substances were accessing SSPs for sterile syringes. People who access SSPs are also less likely to engage in HIV risk behaviors, such as sharing injection equipment, and the use of SSPs has been shown to reduce HIV and HCV transmission and prevent overdose [52,53]. Many SSPs, including those in Baltimore, offer comprehensive services such as safer sex supplies, STI and HBV screening and treatment, overdose education and treatment (naloxone), and linkages to healthcare, including medication-assisted treatment (MAT) [52,54,55,56]. 

It is noteworthy that the other classes, both of which included some injection use, were less associated with obtaining syringes at SSPs. While all classes in the present analysis included at least some injection drug use, other studies have found patterns of polysubstance use that include non-injection drugs use only. Class 2 in this analysis was least likely to inject and was characterized more by non-injection drug use, such as opioid pain relievers and benzodiazepines. This may influence why women in this class were less likely to access SSPs. It may also be that people who inject heroin do so less frequently than those who inject other substances (e.g., cocaine), which are shorter acting and, therefore, used more frequently [57]. However, given the positive outcomes associated with SSPs in other studies, all women who use drugs may benefit from their services. As such, programs may need adaptations to serve people who use other drugs as well such as through providing safe smoking equipment for crack/cocaine use and referrals to MAT and other harm reduction approaches. Similarly, an LCA of SSP users in Florida found heterogeneity in sociodemographic factors and drug use behaviors between classes, indicating a one-size-fits-all approach may not be appropriate [58]. More information is needed to understand why certain subsets of women who inject drugs are less likely to access SSPs. Programming could then be adapted to meet their unique needs.

Another concern is that in this analysis, the poly-route, polysubstance use class was significantly more likely to be non-Hispanic White and younger, while the poly-opioid class had the greatest share of BIPOC. This suggests not only racial differences in drug use patterns but also that SSPs in Baltimore may not be reaching racial and ethnic minority women who sell sex and use drugs. This finding underscores the importance of further examining racial and ethnic disparities related to access to and retention of care for opioid use [59]. The impact of systemic racism cannot be overstated. These racial disparities are especially poignant in Baltimore, which is majority Black and has disproportionately higher HIV incidence and prevalence among BIPOC [60]. Programming to effectively impact the syndemic [61] of HIV, HCV, substance use, poor mental health, poverty, and violence should be tailored to meet the needs of BIPOC women in addition to White women.

The structural risk environment characterized by violence, housing and food insecurity, and sexual risk behaviors, coupled with the syndemic of opioids and poverty among women engaged in street-based transactional sex and who use drugs, may contribute to heightened HIV risk. Harm reduction initiatives and decriminalization of drug use and sex work, which has effectively occurred in Baltimore, are two possible approaches to combat these risks [62]. For women who use drugs and sell sex, these strategies may be essential steps toward addressing these structural vulnerabilities and de-escalating risk. Additional interventions are needed at the societal and structural levels to adequately address the stigma, discrimination, and racism that contribute to the health disparities experienced by women who exchange sex and use drugs [63].

The study has several limitations. First, this study was a secondary data analysis and, as such, was not necessarily powered for the outcomes used in this analysis, which may have affected the results. Second, the analysis did not include alcohol use since, in the baseline survey, alcohol use recency was collected differently than other substance use (past 6 months vs. 3 months). Inclusion of alcohol may have impacted the substance use classes and warrants further research. Third, our survey did not specifically ask about methamphetamine use, though there was an “other: specify” option (in which one participant reported it). In other locations, there are large overlaps between methamphetamine and heroin use. For example, a study found that more than 80% of women who exchange sex in rural West Virginia had recently injected crystal methamphetamine and more than 90% reported heroin injection [64]. Had we asked specifically about methamphetamine use, we may have found a higher prevalence in this population, and this would have likely changed the LCA patterns of polysubstance use. This deserves further study in this population and location. Fourth, our sample only reflects women who exchange sex and use drugs in Baltimore City, a large metropolitan area on the East Coast, and findings may not translate to other populations.

Another limitation is the high missingness in the non-injected heroin plus cocaine variable in the LCA (*n* = 116 missing) due to skip patterns in the survey. While the full information maximum likelihood estimator method uses all available data from each case to estimate missing data using an imputation-like procedure, this may still have impacted the overall class results. Additionally, we were unable to include the transgender women who used drugs in the analysis since their sample size was too small (*n* = 25) and their substance use too different to analyze them as a second group in the LCA. Much more work remains to understand polysubstance use and associated HIV risk factors in transgender women engaged in sex work, especially since they face disproportionally high odds of living with HIV [65].

## 5. Conclusions

This analysis highlights the complex intersection of substance use and structural vulnerabilities among women engaged in street-based transactional sex, particularly the ways in which polysubstance use are associated with HIV risks and vulnerabilities. This population is understudied and underserved but highly vulnerable in the syndemic of HIV/AIDS and other infectious diseases, substance use, violence, and poverty. Evidence-based programs poised to support these women, such as but not limited to SSPs, could benefit from higher and sustained funding and could be better tailored to the unique harm reduction needs of sub-groups of this population, including BIPOC women and women who use drugs but do not inject. Decriminalization of drug use and sex work would further empower these women and allow interventions to better reach people with multiple vulnerabilities, thereby further reducing negative health outcomes.

## Figures and Tables

**Table 1:** Sociodemographic characteristics, drug use indicators, and study outcomes among women engaged in transactional sex in Baltimore City.

Characteristics	Total *n* (%)
Demographics	
Age, mean (sd)	35.8 (8.9)
Race/ethnicity	
BIPOC and Hispanic	80 (32.8)
Non-Hispanic White	164 (67.2)
Education	
Less than high school	127 (52.0)
12th grade or GED	117 (48.0)
Drug use (past 3 months)	
Injection	
Any injection drug use	165 (67.6)
Cocaine	62 (25.4)
Heroin	163 (66.8)
Speedball (heroin and cocaine together)	56 (23.0)
Non-injection	
Cocaine/crack	213 (87.3)
Heroin	104 (42.6)
Heroin + cocaine together (*n* = 116 missing)	22 (9.0)
Opioid pain relievers (*n* = 2 missing)	71 (29.1)
Benzodiazepines	73 (29.9)
Buprenorphine/Suboxone	41 (16.8)
Outcomes (past 3 months)	
Condomless sex with clients (*n* = 1 missing)	98 (40.1)
Food insecurity	134 (54.9)
Housing insecurity	153 (62.7)
Client violence (physical or sexual)	86 (35.3)
Obtained syringes at SSP ^1^ vs. other location	113 (46.3)

^1^ Syringe services program.

**Table 2:** Latent class model fit statistics (*n* = 244).

# Classes	Smallest Class	Log Likelihood	AIC	BIC	Entropy	LMR *p*-Value	Blrt *p*-Value
1	-	−1148.448	2134.896	2346.37	-	-	-
2	73	−1056.847	2151.693	2218.14	0.836	0.000	0.000
3	52	−1031.448	2120.896	2222.314	0.783	0.0016	0.000
4	35	−1015.99	2109.979	2246.369	0.815	0.1544	0.000
5	25	−1001.259	2100.517	2271.897	0.836	0.4855	0.020

**Table 3:** Item–response probabilities of drug use indicators in each latent class among cisgender women (*n* = 244).

	Class 1 Heroin/Cocaine Only Use *n* = 118 (48.4%)		Class 2 Poly-Opioid *n* = 52 (21.3%)		Class 3 Poly-Route, Polysubstance *n* = 74 (30.3%)	
Inj. heroin	0.584	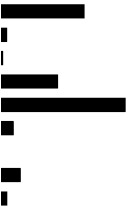	0.389	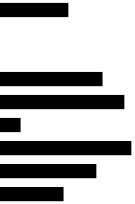	1.000	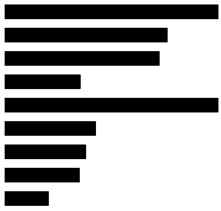
Inj. cocaine	0.042		0.000		0.754	
Inj. speedball	0.015		0.000		0.717	
Heroin	0.397		0.584		0.350	
Cocaine/crack	0.870		0.709		1.000	
Heroin and cocaine	0.089		0.116		0.421	
Opioid pain relievers	0.000		0.749		0.375	
Benzodiazepines	0.137		0.549		0.346	
Bupr./Suboxone	0.044		0.362		0.202	
		0.50		0.50		0.50

**Table 4:** Distribution of sociodemographic characteristics by latent class membership (*n* = 244).

	Class 1	Class 2	2 vs. 1		Class 3	3 vs. 1		3 vs. 2	
	%	%	β	*p*-Value	%	β	*p*-Value	β	*p*-Value
Age (mean)	37.3	35.5	−0.033	0.181	34.0	−0.041	**0.038**	−0.008	0.746
Race			0.851	**0.046**		−0.239	0.546	−1.09	**0.012**
BIPOC and Hispanic	30.8	48.2			23.9				
White, non-Hispanic	69.2	51.8			76.1				
Education < HS	51.1	45.4	−0.316	0.452	58.4	0.240	0.481	0.557	0.187

Bold indicates significance at the *p* < 0.05 level.

## Data Availability

Deidentified participant data are available from S.G.S. at Johns Hopkins University (ORCID: 0000-0001-8399-7544) on reasonable request and review after submission of a manuscript concept form.
